# Li_*x*_@C_60_: Calculations of the Encapsulation Energetics and Thermodynamics

**DOI:** 10.3390/ijms9091841

**Published:** 2008-09-17

**Authors:** Zdeněk Slanina, Filip Uhlík, Shyi-Long Lee, Ludwik Adamowicz, Shigeru Nagase

**Affiliations:** 1 Department of Theoretical and Computational Molecular Science, Institute for Molecular Science, Myodaiji, Okazaki 444-8585, Aichi, Japan; 2 School of Science, Charles University, 128 43 Prague 2, Czech Republic; 3 Department of Chemistry and Biochemistry, National Chung-Cheng University, Chia-Yi 62117, Taiwan; 4 Department of Chemistry, University of Arizona, Tucson, AZ 85721-0041, USA

**Keywords:** endohedral fullerenes, calculated energetics and thermodynamics, structure and bonding, metallofullerene stabilities, computational optimization of syntheses

## Abstract

Li@C_60_ and Li@C_70_ can be prepared and thus, their calculations at higher levels of theory are also of interest. In the report, the computations are carried out on Li@C_60_, Li_2_@C_60_ and Li_3_@C_60_ with the B3LYP density-functional theory treatment in the standard 3-21G and 6-31G* basis sets. The computed energetics suggests that Li*_x_* @C_60_ species may be produced for a few small *x* values if the Li pressure is enhanced sufficiently. In order to check the suggestion, a deeper computational evaluation of the encapsulation thermodynamics is carried out.

## 1. Introduction

There has been a renewed interest [1–20] in systems containing alkali metals and fullerenes, in particular Li@C_60_ and Li@C_70_ produced by low energy ion implantation [11,13,14] in bulk amounts. The vibrational spectra were obtained [13,14] for Li@C_60_ and Li@C_70_. Li_2_@C_60_ was also evidenced in observations [11]. Similarly, for example, Ca@C_74_, Sr@C_74_, and Ba@C_74_ can be prepared by high-temperature techniques [21–24]. This experimental progress makes calculations of the species even more interesting. In the report, the calculations are carried out on Li@C_60_, Li_2_@C_60_, and Li_3_@C_60_, using the density-functional theory (DFT) treatments. Both potential energy and Gibbs free energy terms are evaluated.

## 2. Calculations

The geometry optimizations were carried out with Becke’s three parameter functional [25] with the non-local Lee-Yang-Parr correlation functional [26] (B3LYP) in the standard 3-21G basis set (B3LYP/3-21G). The geometry optimizations were performed with the analytically constructed energy gradient as implemented in the Gaussian program package [27].

In the optimized B3LYP/3-21G geometries, the harmonic vibrational analysis was carried out with the analytical force-constant matrix. In the same optimized geometries, higher-level single-point energy calculations were also performed, using the standard 6-31G* basis set, i.e., the B3LYP/6-31G* level (or, more precisely, B3LYP/6-31G*//B3LYP/3-21G). As Li@C_60_ and Li_3_@C_60_ are radicals, their computations were carried out using the unrestricted B3LYP treatment for open shell systems (UB3LYP). The ultrafine integration grid was used for the DFT numerical integrations throughout.

## 3. Results and discussion

**Figure 1.**
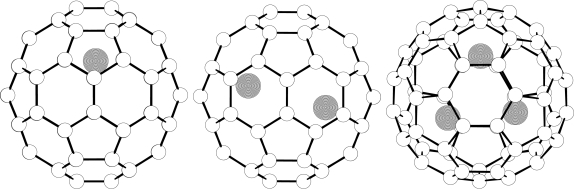
B3LYP/3-21G optimized structures of Li*_x_*@C_60_ (the Li atoms are darkened).

The UB3LYP approach is preferred here over the restricted open-shell ones (ROB3LYP) as the latter frequently exhibits a slow SCF convergency or even divergency. Although the unrestricted Hartree-Fock (UHF) approach can be faster, it can also be influenced by the so called spin contamination [28] and indeed, this factor was an issue in our previous [15] UHF SCF calculations as the UHF/3-21G spin contamination turned out to be higher than recommended threshold [28] in the expectation value for the 〈*S*^2^〉 term where *S* stands for the total spin. As long as the deviations from the theoretical value are smaller than 10%, the unrestricted results are considered applicable [28]. This requirement is well satisfied for the Li@C_60_ and Li_3_@C_60_ species. Fig. 1 shows the computed structures of Li@C_60_, Li_2_@C_60_, and Li_3_@C_60_. In all the three cases the Li atoms in the optimized structures are shifted from the cage center towards its wall. In particular, in the Li@C_60_ species the shortest computed Li-C distance is 2.26 Å, while in a central location (optimized as a stationary point) the shortest Li-C distance at the UB3LYP/3-21G level is 3.49 Å. As for the energetics of the centric and off-centric structure, the central location is placed by some 9.9 kcal/mol higher at the UB3LYP/3-21G level. However, the energy separation is further increased in the UB3LYP/6-31G*//UB3LYP/3-21G treatment, namely to 15.0 kcal/mol. The metal atom in the off-centric Li@C_60_ species is localized above a C-C bond shared by pentagon and hexagon (though an alternative description as above hexagon would also be possible). However, the system does not exhibit any symmetry. Distortion of the cage can be seen from the rotational constants. The icosahedral C_60_ cage at the B3LYP/3-21G level has one uniform rotational constant of 0:0833 GHz. If in the UB3LYP/3-21G optimized Li@C_60_ species the metal atom is removed, the remaining distorted C_60_ cage has the rotational constants 0.0832, 0.0830, and 0:0829 GHz. The distorted cage is higher in energy compared to the icosahedral cage by about 2.5 kcal/mol at the B3LYP/3-21G level.

In the Li_2_@C_60_ case (approximative description as location above hexagon), the shortest Li-C distance is even bit shorter, 2.14 Å. Interestingly enough, Li_2_@C_60_ exhibits center of symmetry. The Li-Li separation is computed as 3.29 Å, i.e., substantially longer than the observed value in the free (neutral) Li_2_ molecule (2.67 Å, cf. refs. [29–31]) – obviously an effect of the positive charges on the encapsulated atoms. In the Li_3_@C_60_ species (with approximative description as localization above C-C bonds shared by pentagon and hexagon), the shortest computed Li-C contact is even further reduced to 2.05 Å. The Li-Li distances in the encapsulated Li_3_ cluster are not equal – they are computed as 2.70, 2.76 and 2.84Å. Incidentally, while the observed Li-Li distance for free Li_2_ is [29–31] 2.67 Å, the B3LYP/3-21G computed value is 2.725 Å (it changes to 2.723 Å at the B3LYP/6-31G* level). Similarly, also the observed values for the free Li_3_ cluster are available [32,33], actually for two triangular forms – opened (2.73, 2.73, 3.21 Å) and closed (3.05, 3.05, 2.58 Å). The UB3LYP/3-21G computed distances in the free Li_3_ opened cluster are 2.78, 2.78, and 3.30 Å. Hence, there is a good theory-experiment agreement. The B3LYP/3-21G formal Mulliken charge (the largest value) found on the Li atoms is somewhat decreasing in the Li@C_60_, Li_2_@C_60_, and Li_3_@C_60_ series with the values of 1.16, 1.10, and 0.86, respectively (the charges are somewhat reduced at the B3LYP/6-31G* level). Nevertheless, the total charge transferred to the cage is increasing in the series: 1.16, 2.21, and 2.46 Å.

The vibrational analysis enables to test if a true local energy minimum was found. All the computed frequencies for the structures in Fig. 1 are indeed real and none imaginary (though we could also locate some saddle points not discussed here). The lowest computed vibrational frequencies are mostly represented by motions of the Li atoms. Obviously, owing to symmetry reductions upon encapsulation, the symmetry selection rules do not operate any more in the way they simplify the C_60_ vibrational spectra [34]. Hence, the vibrational spectra of Li*_x_*@C_60_ must be considerably more complex than for the icosahedral (empty) C_60_ cage with just four bands in its IR spectrum [34]. This increased spectral complexity has indeed been observed [13,14]. Incidentally, the observed harmonic frequency [29–31] for free Li_2_ is 351 cm^−1^ while the computed B3LYP/3-21G term is 349 cm^−1^ (and the B3LYP/6-31G* value 342 cm^−1^). For the endohedrals, larger-basis frequency calculations are not yet common.

There is a general stability problem related to fullerenes and metallofullerenes – either the absolute stability of the species or the relative stabilities of clusters with different stoichiometries. One can consider an overall stoichiometry of a metallofullerene formation:

(1) $$x\text{Y(}g\text{)}\;+\;{\text{C}}_{n}(g)={\text{Y}}_{x}@{\text{C}}_{n}(g).$$

The encapsulation process is thermodynamically characterized by the standard changes of, for example, enthalpy $\Delta H_{{\text{Y}}_{x}@{\text{C}}_{n}}^{○}$ or the Gibbs energy $\Delta G_{{\text{Y}}_{x}@{\text{C}}_{n}}^{○}$. In a first approximation, we can just consider the encapsulation potential-energy changes Δ*E*_Y_*x*_@C_*n*__. Table 1 presents their values for Li*_x_*@C_60_. The absolute values increase with the increasing number of the encapsulated Li atoms. In order to have some directly comparable relative terms, it is convenient to consider the reduced Δ*E*_Y_*x*_@C_*n*__/*x* terms related to one Li atom. The absolute values of the reduced term decrease with increasing Li content, nevertheless, the decrease is not particularly fast (so that, a further increase of the number of encapsulated Li atoms could still be possible). The computational findings help to rationalize why also the Li_2_@C_60_ endohedral could be observed [11]. Although the basis set superposition error is not estimated for the presented values (an application of the Boys-Bernardi counterpoise method may be somewhat questionable in this situation), the correction terms could be to some extent additive. Interestingly enough, the stabilization of metallofullerenes is mostly electrostatic as documented [35,36] using the topological concept of ‘atoms in molecules’ (AIM) [37,38] which shows that the metal-cage interactions form ionic (and not covalent) bonds.

Let us further analyze the encapsulation series from eq. 1. As already mentioned, the encapsulation process is thermodynamically characterized by the standard changes of enthalpy $\Delta H_{{\text{Y}}_{x}@{\text{C}}_{n}}^{○}$ or the Gibbs energy. $\Delta G_{{\text{Y}}_{x}@{\text{C}}_{n}}^{○}$. The thermodynamic functions are calculated here using the standard partition functions available in the Gaussian program package [27], i.e., in the rigid rotor and harmonic oscillator approximation. The equilibrium composition of the reaction mixture is controlled by the encapsulation equilibrium constants *K*_Y_*x*_@C_*n*_,*p*_

(2) $$K_{{\text{Y}}_{x}@{\text{C}}_{n},p}=\frac{p_{{\text{Y}}_{x}@{\text{C}}_{n}}}{p_{\text{Y}}^{x}p_{C_{n}}}$$

expressed in the terms of partial pressures of the components. The encapsulation equilibrium constants are interrelated with the the standard encapsulation Gibbs energy change:

(3) $$\Delta G_{{\text{Y}}_{x}@{\text{C}}_{n}}^{\text{○}}=-RT\text{log}K_{{\text{Y}}_{x}@{\text{C}}_{n,p}}.$$

Temperature dependency of the encapsulation equilibrium constant *K*_Y_*x*_@C_*n*_,*p*_ is then described by the van’t Hoff equation:

(4) $$\frac{d\text{log}K_{Y_{x}@{\text{C}}_{n},p}}{dT}=\frac{\Delta H_{{\text{Y}}_{x}@{\text{C}}_{n}}^{○}}{RT^{2}}$$

where the $\Delta H_{{\text{Y}}_{x}@{\text{C}}_{n}}^{○}$ term is typically negative so that the encapsulation equilibrium constants decrease with increasing temperature.

Let us further suppose that the metal pressure *p*_Y_ is actually close to the respective saturated pressure *p_Y;sat_*. While the saturated pressures *p_Y;sat_* for various metals are known from observations [39], the partial pressure of C*_n_* is less clear as it is obviously influenced by a larger set of processes (though, *p* C*_n_* should exhibit a temperature maximum and then vanish). Therefore, we avoid the latter pressure in our considerations at this stage. As already mentioned, the computed equilibrium constants *K*_Y_*x*_@C_*n*_,*p*_ have to show a temperature decrease with respect to the van’t Hoff equation (4). However, if we consider the combined $p_{\text{Y},sat}^{x}K_{{\text{Y}}_{x}@C_{n,p}}$ terms

(5) $$p_{{\text{Y}}_{x}@{\text{C}}_{n}}∼p_{\text{Y},sat}^{x}K_{{\text{Y}}_{x}@{\text{C}}_{n},p},$$

that directly control the partial pressures of the Y*_x_*@C*_n_* encapsulates in an encapsulation series (based on one common C*_n_* fullerene), we get a different picture. The considered $p_{\text{Y},sat}^{x}K_{\text{X}@{\text{C}}_{n,p}}$ term can frequently (though not necessarily) be increasing with temperature so that a temperature enhancement of metallofullerene formation in the electric-arc technique would be still possible. An optimal production temperature could be evaluated in a more complex model that also includes temperature development of the empty-fullerene partial pressure.

If we however want to evaluate production abundances in a series of metallofullerenes like Li@C_60_, Li_2_@C_60_ and Li_3_@C_60_, just the $p_{Y,sat}^{x}K_{Y_{x}@C_{n,p}}$ product terms can straightforwardly be used. The rigidrotor and harmonic-oscillator partition functions and entropy terms are evaluated at the B3LYP/3-21G level, the potential-energy change at the B3LYP/6-31G* level. The results in Table 2 show several interesting features. For all three members of the series – Li@C_60_, Li_2_@C_60_ and Li_3_@C_60_ – the $p_{\text{Y},sat}^{x}K_{{\text{Y}}_{x}@{\text{C}}_{n,p}}\;$ quotient increases with temperature. This behavior results from a competition between the decreasing encapsulation equilibrium constants and increasing saturated metal pressure.

In order to allow for cancellation of various factors introduced by the computational approximations involved, it is better to deal with the relative quotient $\frac{p_{\text{Y},sat}^{x}K_{{\text{Y}}_{x}@{\text{C}}_{n,p}}\;}{p_{\text{Y},sat}K_{\text{Y}@{\text{C}}_{n,p}}\;}$. Table 2 shows that the production yield of Li_2_@C_60_ in the high-temperature synthesis should be by at least four orders of magnitude smaller than that of Li@C_60_. Chances for production of Li_3_@C_60_ should be still by at least two orders of magnitude worse compared to Li_2_@C_60_. Interestingly enough, an endohedral with a relatively lower value of the encapsulation equilibrium constant could, in principle, still be produced in larger yields if a convenient over-compensation by higher saturated metal pressure can take place owing to the exponent in the pressure in term (5). In fact, we are dealing with a special case of clustering under saturation conditions [40]. The saturation regime is a useful simplification – it is well defined, however, it is not necessarily always achieved. Under some experimental arrangements, under-saturated or perhaps super-saturated metal vapors are also possible. This reservation is applicable not only to the electric-arc treatment but even more likely with the low energy ion implantation [11,13,14]. Still, eqs. (2) and (5) remain valid, however, the metal pressure has to be described by the values actually relevant. For some volatile metals their critical temperature can even be overcome and the saturation region thus abandoned.

Although the energy terms are likely still not precise enough, their errors could be comparable in the series and thus, they should cancel out in the relative terms. Therefore, the suggested relative terms should be rather reliable values. This cancellation could also be the case of other terms involved like the basis set superposition error important for evaluation of the encapsulation potential-energy changes. Another term that should still be evaluated is the electronic partition function as low-lying electronic excited states can make significant contributions into thermodynamics at high temperatures [41]. Finally, a cancellation in the relative terms should also operate for the higher corrections to the rigid-rotor and harmonic-oscillator partition functions, including motions of the encapsulate. The motion of the endohedral atom is highly anharmonic, however, its description is yet possible only with simple potential functions. It has been known from computations and NMR observations [42] that the encapsulated atoms can exercise large amplitude motions, especially so at elevated temperatures (unless the motions are restricted by cage derivatizations [43]). Therefore, in the NMR observations metallofullerenes usually exhibit the highest (topologically) possible symmetry which reflects averaging effects of the large amplitude motions (for this reason, also the symmetry numbers of the Li endohedrals in this paper were taken [44] as 60). As long as we are interested in the relative production yields, the anharmonic effects should at least to some extent be cancelled out in the relative quotient as also demonstrated [19] in some model calculations. Thus, the calculated relative production yields suggested in this study should be reasonably applicable to a broader spectrum of endohedral systems [45].

## 4. Conclusions

Calculations of Li@C_60_, Li_2_@C_60_ and Li_3_@C_60_ with the B3LYP density-functional theory treatment in the standard 3-21G and 6-31G* basis sets have been combined with evaluations of the encapsulation thermodynamics. The production yield of Li_2_@C_60_ in the high-temperature synthesis should be by at least four orders of magnitude smaller compared to Li@C_60_ while that of Li_3_@C_60_ should be still by at least two orders of magnitude lower compared to Li_2_@C_60_. The suggested evaluation of the relative populations is actually applicable to endohedrals in general.

## Figures and Tables

**Table 1.** Computed encapsulation potential-energy changes Δ*E*_Y*_x_*@C*_n_*_ (kcal/mol) for Li*_x_*@C_60_ at the B3LYP/6-31G*//B3LYP/3-21G level.

Species	Δ*E*_Y*_x_*@C*_n_*_	Δ*E*_Y*_x_*@C*_n_*_/*x*
Li@C_60_	−28.4	−28.4
Li_2_@C_60_	−51.1	−25.6
Li_3_@C_60_	−71.0	−23.7

**Table 2.** The products of the encapsulation equilibrium constants *K*_Y*_x_*@C_*n*_,*p*_ with the related metal saturated-vapor pressures [39] *p_Y;sat_* for Li@C_60_, Li_2_@C_60_, and Li_3_@C_60_ computed for selected illustrative temperatures *T*. The potential-energy change is evaluated at the B3LYP/6-31G^*^ level and the entropy part at the B3LYP/3-21G level; the standard state is ideal gas phase at 101325 Pa pressure.

*T* (K)	*K_Y_x_@C_n_,p_* (atm^−*x*^)	*p_Y,sat_* (atm)	$p_{Y,sat}^{x}K_{Y_{x}@C_{n,}p}$	$\frac{p_{Y,sat}^{x}K_{Y_{x}@C_{n,}p}}{p_{Y,sat}K_{Y@C_{n,}p}}$
Li@C_60_				
298.15	6.62×10^17^	3.52×10^−23^	2.33×10^−5^	1.0
1000	3.47×10^2^	9.72×10^−4^	0.337	1.0
1500	3.05	0.467	1.42	1.0
2000	0.305	10.1	3.08	1.0
Li_2_@C_60_				
298.15	2.62×10^27^	3.52×10^−23^	3.24×10^−18^	1.39×10^−13^
1000	3.30	9.72×10^−4^	3.11×10^−6^	9.24×10^−6^
1500	7.47×10^−4^	0.467	1.63×10^−4^	1.15×10^−4^
2000	1.26×10^−5^	10.1	1.29×10^−3^	4.18×10^−4^
Li_3_@C_60_				
298.15	2.42×10^36^	3.52×10^−23^	1.05×10^−31^	4.52×10^−27^
1000	0.282	9.72×10^−4^	2.58×10^−10^	7.67×10^−10^
1500	2.72×10^−6^	0.467	2.77×10^−7^	1.95×10^−7^
2000	9.90×10^−9^	10.1	1.02×10^−5^	3.31×10^−6^
